# Optomechanics of a stable diffractive axicon light sail

**DOI:** 10.1140/epjp/s13360-020-00542-1

**Published:** 2020-07-14

**Authors:** Prateek R. Srivastava, Grover A. Swartzlander

**Affiliations:** Chester F. Carlson Center for Imaging Science, Rochester Institute of Technology, Rochester, NY 14623 USA

## Abstract

Beamed propulsion of a light sail based on radiation pressure benefits from a passively self-stabilizing “beam riding” diffractive film. We describe the optomechanics of a rigid non-spinning light sail that mitigates catastrophic sail walk-off and tumbling by use of a flat axicon diffraction grating. A linear stability analysis and numerical integration of the coupled translational and rotational equations of motion are examined. Stability is traded against longitudinal acceleration. The examined system achieves 90% of the theoretical longitudinal force limit and stability against a relative sail translation up to 30% of the sail radius when the payload is attached to a long boom.

## Introduction

Optical momentum carried by light [1] may be redirected by means of reflection, diffraction, scattering, or absorption to achieve radiation pressure on a material body. The idea of propelling a spacecraft to high velocities using solar radiations was first proposed in the early Twentieth century by Tsiolkovsky [2]. With the advent of lasers in the 1960s, laser-propelled sails reaching relativistic velocities for interstellar travel were proposed [3–13]. One of the many challenges associated with a laser-propelled light sail is the achievement of “beam-riding,” i.e., the autonomous ability to remain in the beam path without tumbling or sliding out. A decades-long approach considers shaped mirror structures [14–24]. The ponderomotive (or gradient) force as used in optical tweezers [25] is currently negligible for practical space systems. The proposed use of diffraction to impart optical momentum to a body [26] provides an opportunity to decouple the sail shape from the momentum transfer process, thereby affording a new degree of design latitude. For example, a one-dimensional bi-grating has been explored to demonstrate the principle of self-stability [27, 28]. The first experimental verification of this principle, along with the measurements of parametric damping, was reported by Chu [29, 30]. Furthermore, advancements in the design and fabrication of diffractive films using metamaterial principles provide opportunities to engineer desired optomechanical and other properties into the functionality of a sail [31–44]. In the near term we envision the integration of diffractive light sail components on future solar sailing missions to help resolve engineering challenges [45] such as attitude control (see for example, Near-Earth Asteroid Scout [46], Solar Polar Imager [47], and Solar Cruiser [48]).

This report extends our one-dimensional theoretical investigations of a bi-grating to a two-dimensional axicon grating sail. Section 2 describes the transfer of momentum from the light beam to the sail, making use of sail and observer reference frames moving at small relative velocities, so that the Doppler-shifted wavelength may be ignored. The equations of motion for linear and angular degrees of freedom owing to optomechanical force and torque are described in Sect. 3. A linear stability analysis is described in Sect. 4 where conditions for stable light propulsion are described. Numerical solutions of the equations of motion are presented in Sect. 5, including an analysis of motion in the stable regime. Important findings are summarized in Sect. 6.

## Photon momentum transfer to a diffractive sail

**Fig. 1:**
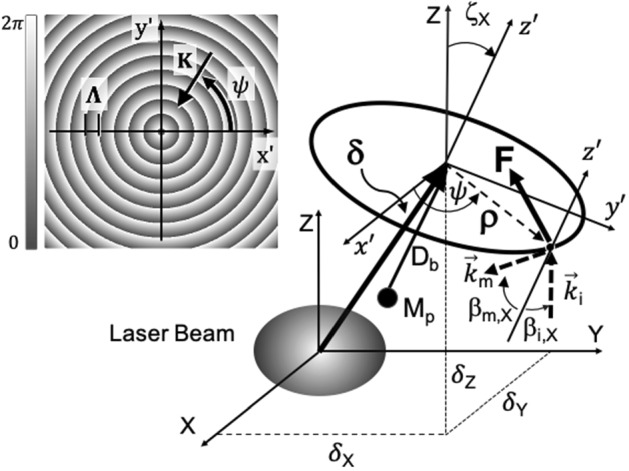
Diffractive sail illuminated by a beam at incident angle $\beta _{i,X}$ and diffraction angle $\beta _{m,X}$. Sail tilt axis $\zeta _X = - \beta _{i,X}$. Attached payload of mass $M_p$ with boom length $D_b$. Laser beam origin $(X,Y,Z)=(0,0,0)$. Sail displacement $\pmb {\vec {\delta }}$. Inset: magnified view of axicon phase with grating vector $\vec {K}$

Let us consider a laser beam of characteristic radial width *w* incident upon a sail of radius *a*. Radiation pressure applies a local force at all sail points, resulting in longitudinal acceleration along the optical axis, lateral force, and torque. The minimum beam size, $w_0$ (the waist), is positioned at the origin of the observer coordinate system (*X*, *Y*, *Z*), as illustrated in Fig. 1, and the beam propagates in the *Z*-direction (the optical axis). The electric field profile of a monochromatic beam of wavelength $\lambda$ and constant power *P* may be expressed [49]

1 $$\begin{aligned} E(X,Y,Z)= & {} \sqrt{I_0(Z)}\ (w_0/w(Z)) \ \mathrm {exp}\left( -(X^2 + Y^2)/w(Z)^2\right) \ \mathrm {exp}\left( i\Phi (r,z)\right) \nonumber \\&\times \mathrm {exp}\left( i\left( k_Z Z - \omega t \right) \right) \end{aligned}$$

where $k_Z = 2\pi /\lambda$, $\omega = ck$ is the angular frequency, *c* is the speed of light, $I_0(Z) = 2P/\pi w^2(Z)$ is the irradiance on the optical axis, $w(Z) = w_0 \left[ 1 + (Z/Z_0)^2\right] ^{1/2}$ is the radial beam size, $Z_0 = \pi w_0^2 / \lambda$ is the diffraction length, $\Phi (r,z) =k_z (X^2 + Y^2)/2R(z) -\mathrm {arctan}(Z/Z_0)$, and $R(Z) = Z\left[ 1 + (Z_0/Z)^2\right]$ for a $\mathrm {TEM}_{00}$ Gaussian beam. Assuming the beam is much larger than the wavelength $(w_0>> \lambda )$, we ignore the transverse component of the wave vector, $k_X = \partial \Phi / \partial X$ and $k_Y = \partial \Phi / \partial Y$, which are much smaller than $k_Z$. That is, the paraxial approximation is made such the incident wave vector may be expressed $\vec {k}_i = (2 \pi / \lambda ) \hat{Z}$.

We consider a sail comprised of a reflection grating that diffracts light toward the sail axis when illuminated at normal incidence. That is, the sail functions as an optical axicon (see inset of Fig. 1), having a periodic phase profile, $$\Phi _{axicon} (\rho '+\Lambda ) = $$$$\Phi _{axicon} (\rho ') = $$$$- 2 \pi (\rho '/\Lambda )$$ where $$\rho ' = (x'^2 + y'^2)^{1/2}$$. For analytical convenience we assume a single diffraction order, noting that this analysis may be readily extended to include multiple reflection and transmission orders. The axicon grating vector $$\vec {K}$$ lies in the plane of the sail and points radially toward the sail axis (see inset of Fig. 1).

The grating vector (see inset of Fig. 1) of the sail is directed radially inward from the center of the sail and is expressed

2 $$\begin{aligned} \vec {K} = -(2\pi /\Lambda )\left( \cos \psi \ \hat{x}' + \sin \psi \ \hat{y}'\right) \end{aligned}$$

where $\Lambda$ is the grating period and $\psi$ is the polar angle measured counterclockwise from $\hat{x}'$. At normal incidence, the angle between $\hat{Z}$ and $\hat{z}$ is zero, i.e., the sail normal and incident wave vector are perfectly aligned and the grating functions as a reflective axicon.

For an arbitrary attitude, the momentum imparted to the sail may be determined from the difference of linear photon momenta before and after diffraction. This difference is quantified by the photon momentum transfer efficiencies in the two reference frames:

3a $$\begin{aligned}&\vec {\eta }' = \left( \vec {k}'_{i} - \vec {k}'_{d} \right) / \left( 2 \pi / \lambda \right) \end{aligned}$$

3b $$\begin{aligned}&\vec {\eta } = \left( \vec {k}_{i} - \vec {k}_{d} \right) / \left( 2 \pi / \lambda \right) \end{aligned}$$

where $\vec {k}'_i$ ($\vec {k}_i$) is the incident wave vector in the sail frame (stationary frame) and $\vec {k}'_d$ ($\vec {k}_d$) is the diffracted wave vector in the sail frame (stationary frame). For example, if $\vec {k}_{d} = -\vec {k}_{i} = -(2\pi /\lambda )\hat{Z}$ then $\vec {\eta } = 2\hat{Z}$. We note that for a Doppler-free elastic process $|\vec {\eta }' | = |\vec {\eta }|$. For an arbitrary sail attitude the method of Euler angles is used to relate the wave vectors in the two reference frames (see “Appendix 1”). However, it is instructive to first consider a sail that is tipped in a single direction as depicted in Fig. 1.

Let us therefore set $\zeta _Y = \zeta _Z = 0$ and consider a rotation angle $\zeta _X$ about the $\hat{X}$ axis. The angle $\zeta _X$ represents the attitude of the sail normal ($\hat{z'})$ with respect to the beam axis ($\hat{Z}$) and is measured counterclockwise from $\hat{Z}$, i.e., $\zeta _X < 0$ for the attitude of sail shown in Fig. 1. The angle of incidence $\beta _{i,x}$ is measured counterclockwise from the sail normal such that $\beta _{i,x} = -\zeta _X$ and $\beta _{i,x} > 0$ for the orientation shown in Fig. 1.

In the sail reference frame the incident wave vector may be expressed

4 $$\begin{aligned} {\vec {k}'_{i} = (2\pi /\lambda ) \left( -\sin \beta _{i,X} \ \hat{y}' + \cos \beta _{i,X}\hat{z}'\right) = (2\pi /\lambda ) \left( \sin \zeta _X \ \hat{y}' + \cos \zeta _X \ \hat{z}'\right) \ } \end{aligned}$$

The diffracted wave vector $\vec {k}'_d$ is determined from the phase matching condition, whereby the phase of the electric field tangential to the sail surface is continuous at the interface:

5a $$\begin{aligned}&\vec {k}'_{i} \cdot \hat{x}' = \vec {k}'_{d} \cdot \hat{x}' + m\vec {K} \cdot \hat{x}' \end{aligned}$$

5b $$\begin{aligned}&\vec {k}'_{i} \cdot \hat{y}' = \vec {k}'_{d} \cdot \hat{y}' + m\vec {K} \cdot \hat{y}' \end{aligned}$$

where *m* is the integer-valued diffraction order. For a normally incident beam where $\vec {k}'_i \cdot (\hat{x}' + \hat{y}') = 0$ and $\vec {k}'_d = -m \vec {K}$, the beam is diffracted toward the sail axis as desired and discussed below when $m=-1$.

Let us express the components of the diffracted wave vector by use of a unit vector $\hat{A}$:

6 $$\begin{aligned} \vec {k}'_{d} = (2\pi /\lambda )\left( A_{x'} \ \hat{x}' + A_{y'} \ \hat{y}' + A_{z'} \ \hat{z}'\right) \end{aligned}$$

where phase matching and elastic scattering $(|\vec {k}'_{i}| = |\vec {k}'_{d})|$ provide

7a $$\begin{aligned}&{A_{x'}=-(m\lambda /\Lambda )\cos \psi } \end{aligned}$$

7b $$\begin{aligned}&{A_{y'} = -\sin \beta _{i,X} -(m\lambda /\Lambda ) \sin \psi =\sin \zeta _X -(m\lambda /\Lambda ) \sin \psi } \end{aligned}$$

7c $$\begin{aligned}&A_{z'}=\pm (1- A^2_{x'}-A^2_{y'})^{1/2} \end{aligned}$$

where the − ($+$) sign corresponds to a reflection (transmission) grating. To achieve efficient acceleration along the beam axis we assume a reflection grating in this report.

Let us now describe diffraction in the stationary reference frame where $\vec {k}_{i} = (2 \pi /\lambda ) \hat{Z}$ and

8 $$\begin{aligned} \vec {k}_{d} = (2\pi /\lambda )(B_X\hat{X} + B_Y\hat{Y} + B_Z\hat{Z}) \end{aligned}$$

where the unit vector $\hat{B}$ is the rotated version of $\hat{A}$:

9a $$\begin{aligned}&B_X = A_{x'} \end{aligned}$$

9b $$\begin{aligned}&{B_Y = A_{y'} \cos \beta _{i,X} + A_{z'} \sin \beta _{i,X} = A_{y'} \cos \zeta _X - A_{z'} \sin \zeta _X } \end{aligned}$$

9c $$\begin{aligned}&{B_Z = - A_{y'} \sin \beta _{i,X} + A_{z'} \cos \beta _{i,X} = A_{y'} \sin \zeta _X + A_{z'} \cos \zeta _X } \end{aligned}$$

General expressions relating rotated vectors $\hat{A}$ and $\hat{B}$ are described in “Appendix 2”.

We therefore find the components of the efficiency vectors:

10a $$\begin{aligned}&{\eta _{x'} = (m\lambda /\Lambda )\cos \psi , \quad \quad \quad \quad \quad \quad \quad \eta _{X} = -B_X}\end{aligned}$$

10b $$\begin{aligned}&{\eta _{y'} = (m\lambda /\Lambda )\sin \psi , \quad \quad \quad \quad \quad \quad \quad \eta _{Y} = -B_Y} \end{aligned}$$

10c $$\begin{aligned}&{\eta _{z'} = \cos \beta _{i,X} - A_{z'} = \cos \zeta _X - A_{z'}, \quad \eta _{Z} = 1 - B_Z} \end{aligned}$$

## Optomechanics of a diffractive sail

The force and torque imparted to the sail produce both linear and angular displacements that depend on initial conditions and other factors such as the beam power, sail shape, and the spatial distribution of the grating vector. As depicted in Fig. 1 we assume a rigid circular sail of radius *a* whose distribution in the sail reference frame $\mathscr {F}'$ may be expressed:

11 $$\begin{aligned} P_{\mathscr {F'}}= \mathrm {Circ}\left( \sqrt{{x'}^2 + {y'}^2}/a\right) \end{aligned}$$

where the function $$\mathrm {Circ}(s)$$ has a value of unity (zero) if $${|s| < 1 (|s| > 1}$$). A payload of mass $$M_p$$ is attached to the sail of mass $$M_s$$ by means of a rigid boom of mass $$M_b$$ and length $$D_b$$ and negligible thickness. A positive (negative) value of $$D_b$$ corresponds to a non-exposed (exposed) payload. For convenience we assume $$M_s = M_p$$ such that the center of mass coincides with the mid-point of the boom. For this configuration the principal moment of inertia is $$J_{x'} = J_{y'} = M_sa^2/4 + M_sD_b^2/4 + M_pD_b^2/4 $$ and $${J_{z'} = Ma^2/2}$$ such that the sailcraft has a diagonal inertia tensor $$J=\mathrm {diag}(J_{x'},J_{y'},J_{z'})$$.

An observer standing next to a stationary laser system will observe the sail moving through space in the $$\mathscr {F} =(X,Y,Z)$$ coordinate system, where the reference frame $$\mathscr {F}$$ is described by a right-handed set of unit vectors $$\{ \hat{X}, \hat{Y}, \hat{Z} \}$$ and origin $$\mathscr {O}$$. We wish to predict the position, velocity, and attitude of the sail in that inertial reference frame. However, radiation pressure exerted on the sail is more readily described in the non-inertial reference frame of the sail, $$\mathscr {F}'$$, with right-handed coordinate system $$(x',y',z')$$ and origin $$\mathscr {O}'$$ (see Fig. 1). In a homogeneous coordinate system (see “Appendix 3”), an arbitrary point in $$\mathscr {F}$$$$\left( \mathscr {F}'\right) $$ is expressed as a column vector $${[X,Y,Z,1]^T}$$$$\left( {[x',y',z',1]^T}\right) $$, where the 4th component is a scaling factor set to unity.

Radiation pressure on a sail gives rise to forces and torques that may translate and rotate the sail. The translation of the sail in the frame $\mathscr {F}$ may be described by the displacement vector $\pmb {\delta } = [\delta _X, \delta _Y, \delta _Z]$. We represent the attitude of the sail in this frame in terms of ZYX sequence of Euler angles $\{\zeta _Z, \zeta _Y, \zeta _X \}$ (see “Appendix 1”). For an arbitrary rotation and translation the relationship between the two frames of reference may be expressed

12 $$\begin{aligned} \begin{bmatrix} x'\\ y'\\ z'\\ 1 \end{bmatrix} = \mathbf {H} \begin{bmatrix} X\\ Y\\ Z\\ 1 \end{bmatrix} = \begin{bmatrix} c_Y c_Z &{} c_Y s_Z &{} -s_Y &{} \ \ \ \delta _X \\ s_X s_Y c_Z - c_X s_Z \ \ \ &{} s_X s_Y s_Z + c_X c_Z &{} s_X c_Y &{} \ \ \ \delta _Y\\ c_X s_Y c_Z + s_X s_Z \ \ \ &{} c_X s_Y s_Z - s_X c_Z \ \ \ &{} c_X c_Y &{}\ \ \ \delta _Z\\ 0&{} 0 &{}0 &{} 1 \end{bmatrix} \begin{bmatrix} X\\ Y\\ Z\\ 1 \end{bmatrix} \end{aligned}$$

where ${\mathbf {H}}$ is the Homogeneous transformation matrix described in “Appendix 3”, and the elements containing factors of $c_{X,Y,Z} = \cos \zeta _{X,Y,Z}$ and $s_{X,Y,Z} = \sin \zeta _{X,Y,Z}$ belong to the rotation matrix described in “Appendix 1”.

The net radiation pressure force in the stationary reference frame is found by integrating over the local force elements:

13 $$\begin{aligned} \vec {F}_{net} = (1/c) \iint \limits _{-\infty }^{\infty } I \ P_{\mathscr {F}} \ \cos \phi \ \vec {\eta } \ dX \ dY = M \ddot{\vec {\pmb {\delta }}} \end{aligned}$$

where $P_{\mathscr {F'}}$ is transformed into the reference frame $\mathscr {F}$ by the expression $P_{\mathscr {F}} = \mathbf {H}^{-1} P_{\mathscr {F'}}$, $\phi$ is the angle between the sail normal and the incident wave vector (i.e., $\cos \phi = \hat{Z}\cdot \hat{z}'$), $I = |E(X,Y,Z)|^2$ is the beam irradiance described in Eq. (1), *c* is the speed of light, and we have applied Newton’s second law to the right-hand side where $M=M_s + M_p + M_b$ is the total light sail mass.

Unlike the net force, the net torque $\vec {N'}_{net}$ measured about the center of mass of the sail is calculated in the sail reference frame $\mathscr {F'}$ and may be found by integration:

14 $$\begin{aligned} \vec {N}_{net}' = (1/c) \iint \limits _{-\infty }^{\infty } I \ P_{\mathscr {F'}} \ \cos \phi \ \vec {r}' \times \vec {\eta }' \ dx' dy' \end{aligned}$$

where $\vec {r}' = {x'\hat{x}' + y'\hat{y}' - (D_b/2) \hat{z}'}$ is the moment arm. Euler’s equations for rotational degrees of freedom may be expressed

15 $$\begin{aligned} \begin{aligned} N_{net,x'}&= J_{x'}\dot{\Omega }_{x'} + (J_{z'}-J_{y'})\Omega _{y'}\Omega _{z'} \\ N_{net,y'}&= J_{y'}\dot{\Omega }_{y'} + (J_{x'}-J_{z'})\Omega _{z'}\Omega _{x'} \\ N_{net,z'}&= J_{z'}\dot{\Omega }_{z'} + (J_{y'}-J_{x'})\Omega _{x'}\Omega _{y'} \end{aligned} \end{aligned}$$

where the angular velocity of the sail measured in the reference frame $\mathscr {F}'$ is related to the time rate of change of Euler angles (see “Appendix 1”).

16 $$\begin{aligned} \dot{\vec {\Omega '}} = (\dot{\zeta }_X - s_X\dot{\zeta }_Z ) \ \hat{x}' + ( c_X\dot{\zeta }_Y + c_Y s_X\dot{\zeta }_Z ) \ \hat{y}' + ( - s_X \dot{\zeta }_Y + c_Y c_X\dot{\zeta }_Z ) \ \hat{z}' \end{aligned}$$

and where the dot symbol represents the time derivative. The displacement, velocity, attitude, and angular velocity of the sail may be found by simultaneously solving coupled equations, Eqs. (13)–(16).

## Linear stability analysis of a diffractive sail

From a practical point of view we desire the sail to accelerate in the $$\hat{Z}$$ direction, while otherwise at an equilibrium position centered on the beam and an equilibrium attitude with the sail axis parallel to the optical axis. To determine whether a given set of system parameters satisfies this requirement, linear stability analysis is applied [50]. Let us define a state vector: $$\mathbf {q} =$$$$ [\delta _X, \delta _Y, \zeta _X, \zeta _Y, \dot{\delta }_X, \dot{\delta }_Y, \dot{\Omega }_X, \dot{\Omega }_Y]^T$$. The linearized equations of motion for translation and rotation may be expressed:

17 $$\begin{aligned} {\dot{\mathbf{q}}} = {{\varvec{\Gamma }}}_0 \mathbf {q} =\begin{bmatrix} \dot{\delta }_X\\ \dot{\delta }_Y\\ \dot{\Omega }_X\\ \dot{\Omega }_Y\\ \ddot{\delta }_X\\ \ddot{\delta }_Y\\ \ddot{\Omega }_X\\ \ddot{\Omega }_Y \end{bmatrix} = \begin{bmatrix} 0&{}0 &{}0 &{}0 &{}1 &{}0 &{}0 &{}0 \\ 0&{}0 &{}0 &{}0 &{}0 &{}1 &{}0 &{}0 \\ 0 &{}0 &{}0 &{}0 &{}0 &{}0 &{}1 &{}0 \\ 0 &{}0 &{}0 &{}0 &{}0 &{}0 &{}0 &{}1 \\ \Gamma _{1}&{} \Gamma _{2} &{} 0 &{} 0 &{} 0 &{} 0 &{} 0 &{}0 \\ \Gamma _{3}&{} \Gamma _{4} &{} 0 &{} 0 &{} 0 &{} 0 &{} 0 &{}0 \\ 0 &{}0 &{} \Gamma _{5} &{}\Gamma _{6} &{} 0 &{} 0&{}0 &{} 0\\ 0&{} 0&{} \Gamma _{7} &{} \Gamma _{8}&{} 0&{}0 &{} 0&{}0 \end{bmatrix}_{\mathbf {q_0}} \begin{bmatrix} \delta _X\\ \delta _Y\\ {\zeta _X}\\ {\zeta _Y}\\ \dot{\delta }_X\\ \dot{\delta }_Y\\ \dot{\Omega }_X\\ \dot{\Omega }_Y \end{bmatrix} \end{aligned}$$

where ${{\varvec{\Gamma }}_\mathbf{0}}$ is calculated at the equilibrium state $\mathbf{q}_\mathbf{0} = \mathbf{0}$:

18 $$\begin{aligned} \begin{aligned}&\left. \Gamma _1 = \frac{1}{M}\frac{\partial (F_X/F_0)}{\partial \delta _X}\right| _{\mathbf {q}_\mathbf{0}}, \left. \Gamma _2 = \frac{1}{M}\frac{\partial (F_X/F_0)}{\partial {\zeta _Y}}\right| _{\mathbf {q}_\mathbf{0}}, \left. \Gamma _3 = \frac{1}{J_{y'}}\frac{\partial (N_{y'}/N_0)}{\partial \delta _X}\right| _{\mathbf{q}_\mathbf{0}},\\&\left. \Gamma _4 = \frac{1}{J_{y'}}\frac{\partial (N_{y'}/N_0)}{\partial {\zeta _Y}}\right| _{\mathbf{q}_\mathbf{0}},\\&\left. \Gamma _5 = \frac{1}{M}\frac{\partial (F_Y/F_0)}{\partial \delta _Y}\right| _{\mathbf{q}_\mathbf{0}}, \left. \Gamma _6 = \frac{1}{M}\frac{\partial (F_Y/F_0)}{\partial {\zeta _X}}\right| _{\mathbf{q}_\mathbf{0}}, \left. \Gamma _7 = \frac{1}{J_{x'}}\frac{\partial (N_{x'}/N_0)}{\partial \delta _Y}\right| _{\mathbf {q}_\mathbf{0}},\\&\left. \Gamma _8 = \frac{1}{J_{x'}}\frac{\partial (N_{x'}/N_0)}{\partial {\zeta _X}}\right| _{{\mathbf {q}_\mathbf{0}}} \end{aligned} \end{aligned}$$

By calculating the eigenvalues of the Jacobian of ${\varvec{\Gamma }}_0$, we determine complex frequencies that correspond to state solutions having the time-dependent form $\exp (\gamma _{a,b} t)$, where real values of $\gamma _{a,b}$ provide exponential damping or gain, and imaginary values provide oscillations. Four complex frequencies are found which satisfy:

19a $$\begin{aligned}&\gamma _{a} =\pm \sqrt{\frac{1}{2}\left( \Gamma _{1} + \Gamma _{4} \pm \sqrt{(\Gamma _{1} - \Gamma _{4})^2 + 4\Gamma _{2}\Gamma _{3} }\right) } \equiv \gamma _{a,r} + i \omega _a \end{aligned}$$

19b $$\begin{aligned}&\gamma _{b} =\pm \sqrt{ \frac{1}{2}\left( \Gamma _{5} + \Gamma _{8} \pm \sqrt{(\Gamma _{5} - \Gamma _{8})^2 + 4\Gamma _{6}\Gamma _{7}}\right) } \equiv \gamma _{b,r} + i \omega _b \end{aligned}$$

**Table 1:** List of parameters and values

Parameters	Value
Light Sail	
Grating period, $\Lambda$	1.6 ${\upmu }$m
Diffraction order, *m*	$-1$
Radius, *a*	1.0 m
Mass, $M_s$	0.50 g
Payload mass, $M_p$	0.50 g
Boom length, $D_b$	15.0 m
Boom mass, $M_b$	0.17 g
Total mass, *M*	1.17 g
Moments of inertia, $J_x', J_y'$	$0.06\,\mathrm {kg\,m^{-2}}$
Moments of inertia, $J_z'$	$0.25\,\mathrm {g\,m^{-2}}$
Radius of gyration, $R_g$	7.13 m
Laser beam	
Power, $P_0$	10 kW
Gaussian beam waist, $w_0$	0.5 m
Wavelength, $\lambda$	1.0 ${\upmu }$m
Diffraction length, $Z_0$	$0.79 \times 10^6$m
System	
$\Gamma _1=\Gamma _5$	−0.04 $\mathrm {kg^{-1} m^{-1}}$
$\Gamma _2=\Gamma _6$	0.05 $\mathrm {kg^{-1} rad^{-1}}$
$\Gamma _3=\Gamma _7$	−0.005 $\mathrm {kg^{-1} m^{-3}}$
$\Gamma _4=\Gamma _8$	0 $\mathrm {kg^{-1} m^{-2} rad^{-1}}$
$\omega _0$	0.2 rad/s
$\Delta$	0.157 rad/s
$\omega _l$	0.087 rad/s
$\omega _h$	0.18 rad/s
$T_h$	35 s
$T_l$	72 s
Initial conditions $(t=0)$:	
Displacement, $(\delta _X, \delta _y)$	(0.1 m, −0.1 m)
Attitude, $(\zeta _X, \zeta _Y)$	$(1^\circ , -1^\circ )$
Linear velocity, $(\dot{\delta }_X,\dot{\delta }_Y)$	(0,0)
Angular velocity, $(\dot{\Omega }_X, \dot{\Omega }_Y)$	(0,0)

**Fig. 2:**
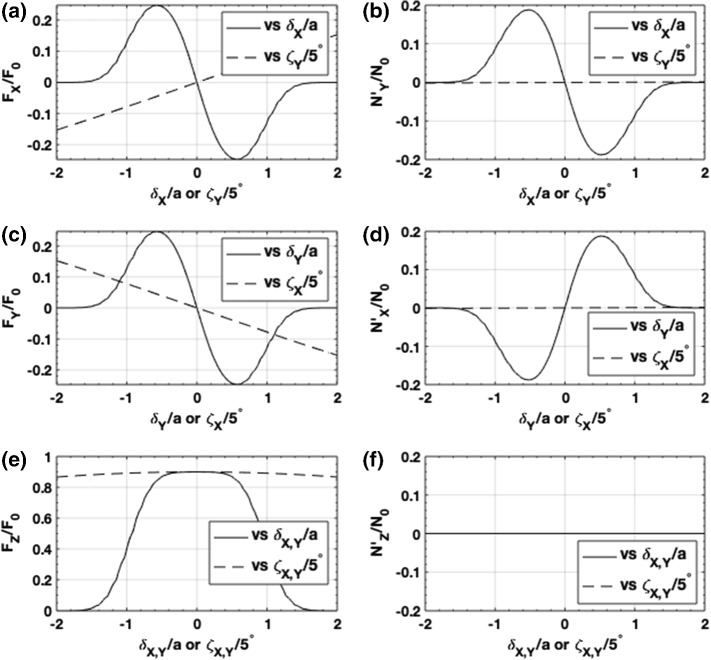
Normalized components of **a, c, e** force and **b, d, f** torque as a function of transverse displacement, $\delta _{X,Y}$, and attitude, $\zeta _{X,Y}$, where $F_0 = 2P_0/c$ and $N_0=F_0D_b/2$

where $\gamma _{a,r}$, $\gamma _{b,r}$, $\omega _a$, $\omega _b$ are real values. The conditions for linear stability are ${\gamma _{a,r}} \le 0$ and ${\gamma _{b,r} \le 0}$, i.e., exponential growth is prohibited. For $\gamma _{a,r} = \gamma _{b,r} = 0$ as found below, the sail oscillates about the equilibrium point with four characteristic periods that depend on system parameters such as the grating period, the size of the sail, the beam size and power, and the moment of inertia of the light sail. What is more, for the symmetric system considered in this report $\Gamma _1 = \Gamma _5 < 0$, $\Gamma _4 = \Gamma _8 = 0$, and $\Gamma _2 \Gamma _3 = \Gamma _6 \Gamma _7 < 0$, $\Gamma _1^2 > 4| \Gamma _2 \Gamma _3 |$ and we therefore find two degenerate frequencies: a high frequency $\omega _h$ and a low frequency $\omega _l$ satisfying

20a $$\begin{aligned}&\omega _h ^2 = (1/2)(\omega _0^2 + \Delta ^2) \end{aligned}$$

20b $$\begin{aligned}&\omega _l ^2 = (1/2)(\omega _0^2 - \Delta ^2) \end{aligned}$$

where $$\omega _0^2 = $$$$\Gamma _1 + \Gamma _4 =$$$$\Gamma _5 + \Gamma _8$$ and $$\Delta ^2 = $$$$((\Gamma _1 - \Gamma _4)^2+4 \Gamma _2 \Gamma _3)^{1/2} = $$$$((\Gamma _5 - \Gamma _8)^2+4 \Gamma _6 \Gamma _7)^{1/2}$$. Therefore we expect the system to display two oscillation modes when excited close to equilibrium.

## Numerical solutions

Closed-form solutions of the system equations of motion generally do not exist, and therefore, numerical integration methods must be applied. For a representative non-optimized case we examined a laser-sail system having parameters listed in Table 1. We assumed a beam power of $P_0 = 10$[kW] (as was used in microwave beam-rider experiments [17, 18]) illuminating a sail of radius $a= 1$[m], with the beam waist $w_0 =0.5$[m] under-filling the sail.

We numerically computed Eqs. (13)–(16) for different initial values of linear and angular displacement, plotting the results in Fig. 2. The linear nature of the force and torque near-equilibrium is clearly evident in Fig. 2 for the range $| \delta _{X,Y}/a | < 0.5$ and $| {\zeta _{X,Y}} | < 2.5^\circ$. We also observe that the force along the beam axis reaches roughly 90% of the maximum theoretical value of $2P_0/c$. Furthermore, the value of the roll torque $N'_z$ is zero, and thus the system does not acquire angular momentum about the sail axis. Changing only the angle ${\zeta _X}$ (${\zeta _Y}$) at equilibrium we also find that the torque $N'_Y$ ($N'_X$) is zero valued.

**Fig. 3:**
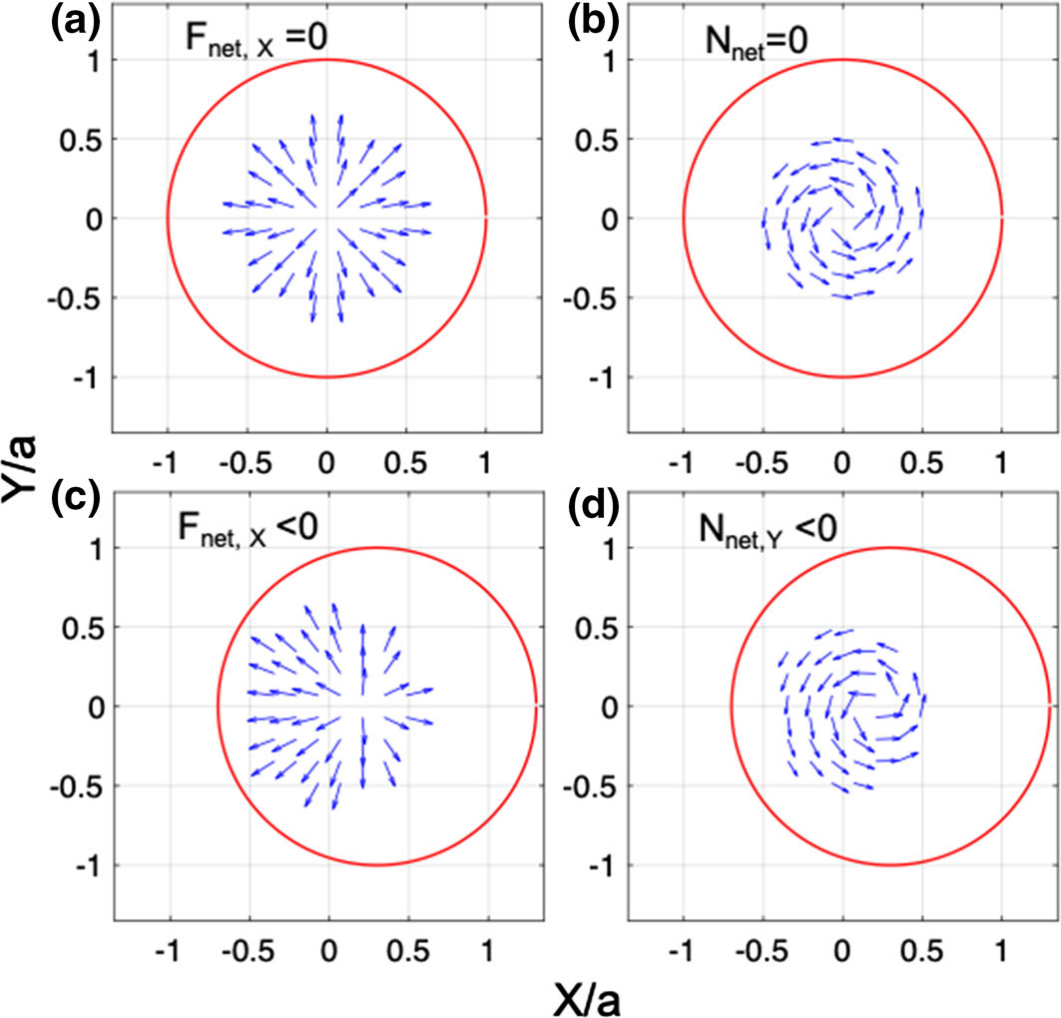
Local forces and torques exerted on an non-tilted axicon sail when the sail and optical axis are **a**, **b** co-linear, and **c**, **d** displaced. **c** The net force drives the sail toward the beam axis $(X,Y) = (0,0)$

A perspective of the net force exerted on the sail at equilibrium is depicted in Fig. 3a where local transverse components of force are displayed, resulting in no net transverse force. Similarly, the net torque exerted on the sail at equilibrium is depicted in Fig. 3b where local transverse components of torque, are displayed, resulting in no net transverse torque. If the sail is displaced from equilibrium to the right, as in Fig. 3c the net force drives the sail to the left. In Fig. 3d the net torque is in the $-\hat{Y}$ direction.

Values of the slopes at the equilibrium points are obtained from Fig. 2, which along with the mass and moments of inertia in Table 1 allow us to determine the values of $\Gamma _j$ (see Eq. (18)). Inserting $\Gamma _j$ into Eq. (20) we find $\omega _h = 0.18$[rad/s] and $\omega _l = 0.087$[rad/s], with respective oscillation periods $T_h = 35$[s] and $T_l = 72$[s]. For a higher power laser beam $\tilde{P}$ we expect proportionally more optomechanical energy to be pumped into the system [28], resulting in higher squared values of frequency and lower squared values of the oscillation periods, $\tilde{T}_{h,l}$. Therefore

21 $$\begin{aligned} \tilde{T}_{h,l} = (P_0 / \tilde{P} )^{1/2} T_{h,l} \end{aligned}$$

For example, if $P = 1$ [GW] the periods are expected to decrease to $T_h = 11$ [ms] and $T_l = 228$ [ms].

**Fig. 4:**
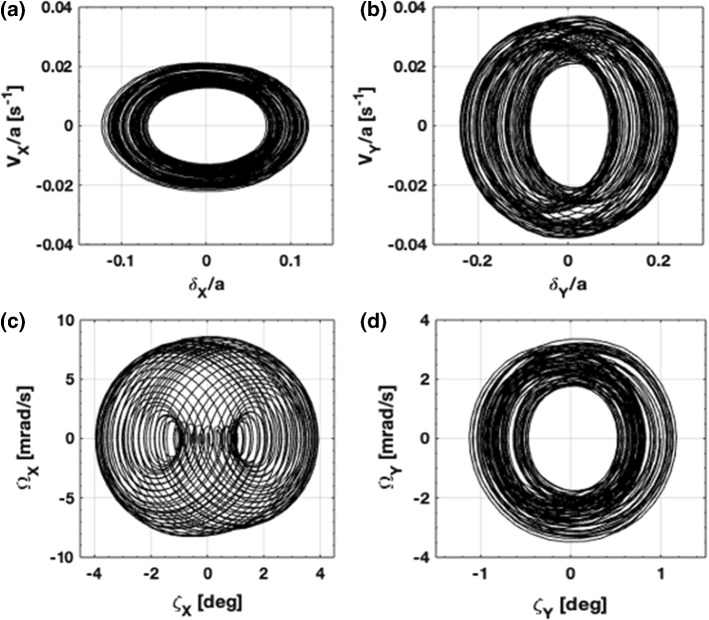
Phase plot for oscillations **a**, **b** along transverse direction and **c**, **d** in attitude about $(\hat{X},\hat{Y})$ is shown, for an initial condition $\pmb {q}=[0.1, -0.1, 1^\circ , -1^\circ , 0,0,0,0]^T$ and time $t = 27{,}400$ [s]

Solutions of the equations of motion for a given set of initial conditions were numerically solved by use of the fourth-order Runge–Kutta method. An example that illustrates stable motion for small perturbations from equilibrium is shown in Fig. 4 for the system initially at rest and displaced: $\delta _X/a = -\delta _Y/a = 0.1$ and ${\zeta _X} = -{\zeta _Y}= 1^\circ$. The phase diagrams correspond to an elapsed time of $t = 780 T_h = 27{,}400\, [s]$. During this time the sail acquires a speed of $\Delta v_Z = 1.4$ [km/s] and traverses a distance of $\Delta Z = 19 \times 10^6$ [km] $= 25 Z_0$, assuming the beam size is controlled, so that it does not overfill the sail. As expected from our linear stability analysis, the system remains stable under this condition. The acceleration $a_Z = 0.51 \mathrm {[m/s^2]}$ may be increased in proportion to the laser power, thereby providing values of $\Delta v_Z$ that are relevant for orbit-changing maneuvers, although the high oscillation frequencies (see above) may become mechanically intolerable if not damped.

**Fig. 5:**
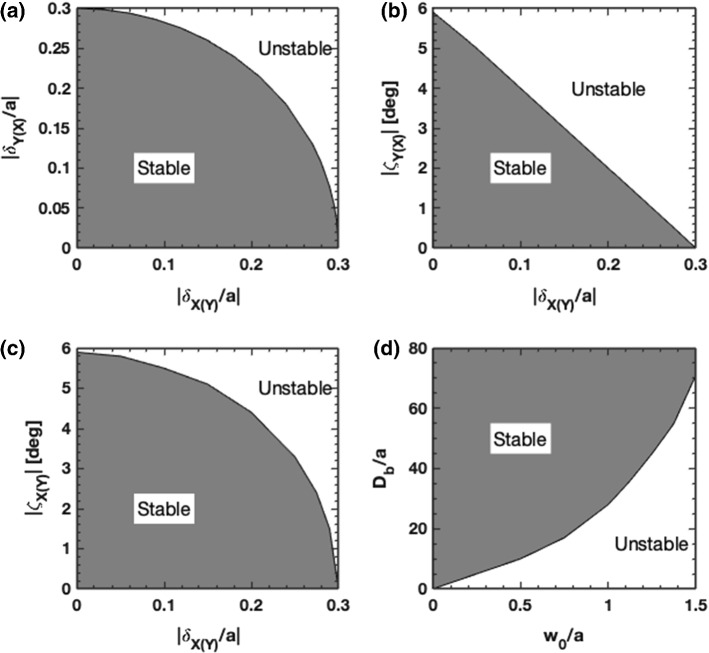
Regions of optomechanical stability for **a** relative linear displacement $\delta _X/a$ versus $\delta _Y/a$, **b** orthogonal linear displacement and attitude axes, $\delta _X/a$ versus ${\zeta _Y}$ (or $\delta _Y/a$ versus ${\zeta _X}$), **c** parallel linear displacement and attitude axes, $\delta _X/a$ versus $\zeta _X$ (or $\delta _Y$ versus $\zeta _Y$) and **d** relative laser beam width versus relative boom length $w_0/a$ versus $D_b/a$

An examination of Fig. 5 indicates that force and torque are nonlinearly related to linear and angular displacements for $| \delta _{X,Y}/a | \gtrsim 0.5$ and $| {\zeta _{X,Y}} | \gtrsim 2.5^\circ$. Below these bounds the system may be characterized by linear and torsional spring models with stiffness values equal to the slopes in Fig. 2. Close to the nonlinear bounds the springs become soft and less able to provide a restoring force or torque. Beyond these bounds the system is driven away from equilibrium. To explore how the departure from linear behavior affects the range of stable motion for the system described in Table 1 we varied the initial conditions across the range $\delta _{X,Y} \in [-a, \, a]$, or ${\zeta _{X,Y}}\in [-10^\circ , \, 10^\circ ]$, with $\dot{\delta }_{X,Y}= \dot{\Omega }_{X,Y}=0$. We then numerically integrated the coupled equations of motion, categorized the observed motion as stable or unstable, and summarized the results in the stability maps shown in Fig. 5. The stable range of linear displacement (assuming ${\zeta _{X,Y}} = \dot{\delta }_{X,Y}= \dot{\Omega }_{X,Y} = 0$ at $t=0$ indicates a stability zone defined by $\delta _X^2 + \delta _Y^2 \le (0.3a)^2$ where the radius 0.3*a* is significantly smaller than the bound $\delta _{X,Y} = 0.5a$. We attribute this smaller zone to the weak force stiffness at 0.3*a* and coupling to motion in other degrees of freedom that do not provide an attraction to equilibrium. A linear zone boundary was found when varying both $\delta _X$ and ${\zeta _Y}$ (with other state parameters equal to zero), and is shown in Fig. 5b. An examination of Fig. 5b indicates that the force at ${\zeta _Y} = 6^\circ$ is equal and opposite to the force at $\delta _X = 0.3a$, suggesting both a reason and an equivalence for the stability boundaries at $\delta _X = 0.3a$ and ${\zeta _Y} = 6^\circ$. The same zone boundary relation was found when varying $\delta _Y$ and $\zeta _X$. According to Fig. 5c the system stability is more robust to simultaneous displacements along and rotations about a common axis. Finally, we explore an example where variations of the boom length and beam size affect stability. In this example, we selected the initial condition: $\delta _X = - \delta _Y = 0.1a$ and ${\zeta _X = - \zeta _Y = 1^\circ }$. As shown in Fig. 5d the system is generally more stable for long boom lengths, but for a given beam size there is a minimum boom length below which the system is unstable. For example, if the beam radius equals half the sail radius, $w_0 = a/2$, as listed in Table 1, we predict a minimum boom length of $D_b = 10 a$. In comparison we made our numerical studies in Section 3 for a boom length of $D_b = 15a$, well into the stable regime. We also predict that stable motion may be achieved when the beam overfills the sail (i.e., $w_0 > a$), but only if the boom length is made significantly larger than the sail radius. For example if $w_0 = a$, stability requires $D_b > 28a$.

## Summary

Diffraction-based light sails provide a design flexibility that is not afforded by reflective sails. This is attributed to the controlled redirection of light by an engineered diffractive surface rather than a deformed reflective surface. We have described the optomechanics of a rigid non-spinning laser-driven sail comprised of a reflective axicon diffraction grating and a payload attached to a boom. A single diffraction order is assumed, producing diffraction toward the optical axis. Such diffraction affords passive stability while also providing longitudinal acceleration along the beam axis. Our example exhibited 90% of the maximum theoretical value of force along the optical axis, with the 10% deficit sacrificed to achieve beam-riding stability against transverse perturbations as large as 30% of the sail radius and attitude perturbations as large as $6^\circ$. Numerical methods were used to integrate the coupled equations of motion, allowing us to illustrate stable oscillations and to map regions of stable and unstable motion. A linear stability analysis predicted as many as four modes of oscillation, reducing to two degenerate frequencies for our symmetric structure. Both the attitude and transverse motion exhibited the two frequencies. The squared frequency was found to increase linearly with beam power. Our optomechanical model may be readily extended to include complex diffractive structures, complex beam shapes, modulated beam power, and a spinning sail. The model requires further work to include the Doppler effect [51], mechanical compliance, and center-of-mass center-of-pressure offsets. Advanced features that may be integrated into the diffractive sail include active attitude control [31, 45, 52].

## Appendix 1: Euler angles

Let $\mathscr {F} = \{ \hat{X}, \hat{Y}, \hat{Z} \}$ represent the reference frame of a stationary coordinate system and $\mathscr {F}' = \{ \hat{x}, \hat{y}, \hat{z} \}$ represent a rotated reference frame. The two frames of reference with orthonormal coordinate systems can be aligned by three consecutive rotations given by Euler angles $\pmb {\zeta } \in \left\{ \zeta _Z, \zeta _Y, \zeta _X\right\}$. As per Euler theorem, two consecutive rotations have to be made about different axis. We make use of the *ZYX* Euler angle sequence with rotation matrices:

22 $$\begin{aligned} \xi _Z = \begin{pmatrix} c_Z &{}\quad s_Z &{}\quad 0 \\ -s_Z &{}\quad c_Z &{}\quad 0\\ 0 &{}\quad 0 &{}\quad 0 \end{pmatrix},\ \ \xi _Y = \begin{pmatrix} c_Y &{}\quad 0 &{}\quad -s_Y \\ 0 &{}\quad 1&{}\quad 0\\ s_Y &{}\quad 0 &{}\quad c_Y \end{pmatrix}, \ \ \xi _X = \begin{pmatrix} 1 &{}\quad 0 &{}\quad 0 \\ 0 &{}\quad c_X &{}\quad s_X\\ 0 &{} -s_X &{} c_X \end{pmatrix} \end{aligned}$$

where $\xi _j$ is the rotation about j-th axis by angle $\zeta _j$ and $c_j = \cos \zeta _j$ and $s_j = \sin \zeta _j$, and $j = X$, *Y*, or *Z*, with the singularity restriction $\zeta _Y \ \in \left( -\pi /2, \pi /2 \right)$ and $\zeta _{X, Z}\ \in \left( 0, 2\pi \right)$. The rotated frame may be expressed $\mathscr {F'} = \pmb {\xi } \mathscr {F}$ where

23 $$\begin{aligned} \pmb {\xi } = \xi _X \xi _Y \xi _Z = \begin{bmatrix} c_Y c_Z &{} c_Y s_Z &{} -s_Y \\ s_X s_Y c_Z - c_X s_Z \ \ \ &{} s_X s_Y s_Z + c_X c_Z &{} s_X c_Y \\ c_X s_Y c_Z + s_X s_Z \ \ \ &{} c_X s_Y s_Z - s_X c_Z \ \ \ &{} c_X c_Y \\ \end{bmatrix} \end{aligned}$$

Conversely we may write $\mathscr {F} = \pmb {\xi }^{-1} \mathscr {F'}$ where

24 $$\begin{aligned} \pmb {\xi }^{-1} = \pmb {\xi }^{T} = \begin{bmatrix} c_Yc_Z &{} \ \ s_Xs_Yc_Z - c_Xs_Z &{} \ \ c_Xs_Yc_Z + s_Xs_Z \\ c_Ys_Z &{} \ \ s_Xs_Ys_Z + c_Xc_Z &{} \ \ c_Xs_Ys_Z - s_Xc_Z \\ -s_Y &{} \ \ s_Xc_Y &{} \ \ c_Xc_Y \end{bmatrix} \end{aligned}$$

The angular velocity $\pmb {\Omega }$ depends on the time rate of change of Euler angles $\pmb {\dot{\zeta }}=[\dot{\zeta }_X,\dot{\zeta }_Y,\dot{\zeta }_Z]^T$ where $\dot{\zeta }_j = d\zeta _j / dt$. For the *ZYX* sequence of Euler angles, the angular velocity in the two frames may be expressed [53]:

25 $$\begin{aligned} \begin{aligned} \pmb {\Omega }' = \pmb {\Omega } = \begin{pmatrix} 1 &{}\quad 0 &{}\quad -s_x\\ 0 &{}\quad c_X &{}\quad c_Y s_X\\ 0 &{}\quad -s_X &{}\quad c_Y c_X \end{pmatrix} \begin{pmatrix} \dot{\zeta }_X\\ \dot{\zeta }_Y\\ \dot{\zeta }_Z \end{pmatrix} \end{aligned} \end{aligned}$$

and the angular velocity vectors are expressed

26a $$\begin{aligned} { \vec {\pmb {\Omega }'} = (\zeta _X -s_X\zeta _Z)\hat{x'} + (c_x\zeta _Y + c_Ys_X\zeta _Z)\hat{y'} + (-s_x\zeta _Y + c_Yc_X\zeta _Z)\hat{z'}} \end{aligned}$$

26b $$\begin{aligned} { \vec {\pmb {\Omega }} = (\zeta _X -s_X\zeta _Z)\hat{X} + (c_x\zeta _Y + c_Ys_X\zeta _Z)\hat{Y} + (-s_x\zeta _Y + c_Yc_X\zeta _Z)\hat{Z}} \end{aligned}$$

## Appendix 2: Rotation of diffraction efficiency vectors

For an arbitrarily orientated sail with respect to a stationary frame, the incident beam, grating vector, and diffracted beam are expressed:

27a $$\begin{aligned}&\vec {k}_i = (2\pi /\lambda )\hat{Z} \ \mathrm {and} \ {\vec {k'_i} = (2\pi /\lambda ) (-s_Y \ \hat{x}' +s_Xc_Y \ \hat{y}' + c_Xc_Y \ \hat{z}')} \end{aligned}$$

27b $$\begin{aligned}&\vec {K} = -(2\pi /\Lambda )(\cos \psi \ \hat{x}' + \sin \psi \ \hat{y}') \end{aligned}$$

27c $$\begin{aligned}&\vec {k'_d} = (2\pi /\lambda )( A_{x'} \ \hat{x}' + A_{y'} \ \hat{y}' + A_{z'} \ \hat{z}') \end{aligned}$$

where

28a $$\begin{aligned}&{A_{x'} = k_{i,x'} - mK_{x'} = -s_Y - (m\lambda /\Lambda )\cos \psi } \end{aligned}$$

28b $$\begin{aligned}&{A_{y'} = k_{i,y'} - mK_{y'} = s_Xc_Y - (m\lambda /\Lambda )\sin \psi }\end{aligned}$$

28c $$\begin{aligned}&A_{z'} = k_{d, z'} = \pm \sqrt{1 - A_{x'}^2 - A_{y'}^2 } \end{aligned}$$

In the stationary frame, the diffracted beam components are expressed

29 $$\begin{aligned} \vec {k}_d = (2\pi /\lambda ) (B_{X} \hat{X} + B_{Y} \hat{Y} + B_{Z} \hat{Z}) \end{aligned}$$

where

30 $$\begin{aligned} \begin{aligned} \begin{bmatrix} B_X\\ B_Y\\ B_Z \end{bmatrix} =\begin{bmatrix} c_Yc_Z &{} \ \ s_Xs_Yc_Z - c_Xs_Z &{} \ \ c_Xs_Yc_Z + s_Xs_Z \\ c_Ys_Z &{} \ \ s_Xs_Ys_Z + c_Xc_Z &{} \ \ c_Xs_Ys_Z - s_Xc_Z \\ -s_Y &{} \ \ s_Xc_Y &{} \ \ c_Xc_Y \end{bmatrix}\begin{bmatrix} A_{x'}\\ A_{y'}\\ A_{z'} \end{bmatrix} = \pmb {\xi }^T \begin{bmatrix} A_{x'}\\ A_{y'}\\ A_{z'} \end{bmatrix} \end{aligned} \end{aligned}$$

The photon momentum transfer efficiency imparted to the sail may be expressed

31 $$\begin{aligned} \vec {\eta }' = \left( \vec {k}'_i - \vec {k}'_d \right) / (2 \pi / \lambda ) \quad \mathrm {and} \quad \vec {\eta } = \left( \vec {k}_i - \vec {k}_d \right) / (2 \pi / \lambda ) \end{aligned}$$

or

32a $$\begin{aligned}&\eta _{x'} = \left( \vec {k}'_\mathrm {i} - \vec {k}'_d \right) / (2 \pi / \lambda ) = (-s_Y - A_{x'}) \end{aligned}$$

32b $$\begin{aligned}&\eta _{y'} = \left( \vec {k}'_i - \vec {k}'_d \right) / (2 \pi / \lambda ) = (s_Xc_Y - A_{y'}) \end{aligned}$$

32c $$\begin{aligned}&\eta _{z'} = \left( \vec {k}'_i - \vec {k}'_d \right) / (2 \pi / \lambda ) = (c_Xc_Y - A_{z'}) \end{aligned}$$

and

33a $$\begin{aligned} \eta _{x} = \left( \vec {k}_i - \vec {k}_d \right) / (2 \pi / \lambda ) = -B_X \end{aligned}$$

33b $$\begin{aligned} \eta _{y} = \left( \vec {k}_i - \vec {k}_d \right) / (2 \pi / \lambda ) = -B_Y \end{aligned}$$

33c $$\begin{aligned} \eta _{z} = \left( \vec {k}_i - \vec {k}_d \right) / (2 \pi / \lambda ) = 1 - B_Z \end{aligned}$$

## Appendix 3: Homogeneous coordinates

Let $[X, Y, Z]^T$ be the Cartesian coordinates of a point in a reference frame $\mathscr {F}$ with origin $\mathscr {O}$ and a right-handed basis $\{ \hat{X}, \hat{Y}, \hat{Z} \}$. The homogeneous coordinates of the point may be expressed [54]

34 $$\begin{aligned} \mathscr {F} = [X,Y,Z,1]^T \end{aligned}$$

Similarly, a second frame $\mathscr {F}'$ with origin $\mathscr {O}'$ and right-handed basis $\{ \hat{x}', \hat{y}', \hat{z}' \}$ is expressed

35 $$\begin{aligned} \mathscr {F}' = [x', y', z', 1]^T \end{aligned}$$

A displacement $\pmb {\delta } = [\delta _X, \delta _Y, \delta _Z]^T$ and rotation $\pmb {\xi }$ of $\mathscr {F}'$ with respect to $\mathscr {F}$ may be written as a matrix operation:

36 $$\begin{aligned} \mathscr {F}'= & {} \mathbf {H} \mathscr {F} \ \ \mathrm {or} \ \ \begin{bmatrix} x'\\ y'\\ z'\\ 1 \end{bmatrix} =\begin{bmatrix} &{} \pmb {\xi } &{} &{} \pmb {\delta } \\ 0&{} 0 &{}0 &{} 1 \end{bmatrix} \begin{bmatrix} X\\ Y\\ Z\\ 1 \end{bmatrix}\nonumber \\= & {} \begin{bmatrix} c_Y c_Z &{} c_Y s_Z &{} -s_Y &{} \ \ \ \delta _X \\ s_X s_Y c_Z - c_X s_Z \ \ \ &{} s_X s_Y s_Z + c_X c_Z &{} s_X c_Y &{} \ \ \ \delta _Y\\ c_X s_Y c_Z + s_X s_Z \ \ \ &{} c_X s_Y s_Z - s_X c_Z \ \ \ &{} c_X c_Y &{} \ \ \delta _Z\\ 0&{} 0 &{}0 &{} 1 \end{bmatrix} \begin{bmatrix} X\\ Y\\ Z\\ 1 \end{bmatrix} \end{aligned}$$

where $\mathbf {H}$ is the homogeneous transformation matrix [55] and ZYX sequence of Euler angles is assumed to calculate the rotation matrix $\pmb {\xi }$ (see Eq. (23)). The inverse mapping from $\mathscr {F'}$ to $\mathscr {F}$ may be expressed:

37 $$\begin{aligned} \mathscr {F} = \mathbf {H}^{-1} \mathscr {F}' \ \ \mathrm {or} \ \ \begin{bmatrix} X\\ Y\\ Z\\ 1 \end{bmatrix} = \mathbf {H}^{-1} \begin{bmatrix} x'\\ y'\\ z'\\ 1 \end{bmatrix}= \begin{bmatrix} &{}\pmb {\xi }^T &{} &{} -\pmb {\xi }^T\pmb {\delta } \\ 0 &{}0 &{}0 &{} 1 \end{bmatrix}\begin{bmatrix} x'\\ y'\\ z'\\ 1 \end{bmatrix} \end{aligned}$$

where $\mathbf {H}^{-1}$ is expressed

38 $$\begin{aligned} \mathbf {H}^{-1}= \begin{bmatrix} c_Yc_Z &{} \ \ s_Xs_Yc_Z - c_Xs_Z &{} \ \ c_Xs_Yc_Z + s_Xs_Z &{}\ \ -(c_Yc_Z)\delta _X - (s_Xs_Yc_Z - c_Xs_Z)\delta _Y - (c_Xs_Yc_Z + s_Xs_Z)\delta _Z \\ c_Ys_Z &{} \ \ s_Xs_Ys_Z + c_Xc_Z &{} \ \ c_Xs_Ys_Z - s_Xc_Z &{}\ \ -(c_Ys_Z)\delta _X - (s_Xs_Ys_Z + c_Xc_Z)\delta _Y - (c_Xs_Ys_Z - s_Xc_Z)\delta _Z \\ -s_Y &{} \ \ s_Xc_Y &{} \ \ c_Xc_Y &{} -(-s_Y)\delta _X - (s_Xc_Y)\delta _Y - (c_Xc_Y)\delta _Z \\ 0 &{} 0 &{} 0 &{} 1 \end{bmatrix}\nonumber \\ \end{aligned}$$
